# General overview on structure prediction of twilight-zone proteins

**DOI:** 10.1186/s12976-015-0014-1

**Published:** 2015-09-04

**Authors:** Bee Yin Khor, Gee Jun Tye, Theam Soon Lim, Yee Siew Choong

**Affiliations:** Institute for Research in Molecular Medicine, Universiti Sains Malaysia, 11800 Minden, Penang Malaysia

## Abstract

Protein structure prediction from amino acid sequence has been one of the most challenging aspects in computational structural biology despite significant progress in recent years showed by critical assessment of protein structure prediction (CASP) experiments. When experimentally determined structures are unavailable, the predictive structures may serve as starting points to study a protein. If the target protein consists of homologous region, high-resolution (typically <1.5 Å) model can be built via comparative modelling. However, when confronted with low sequence similarity of the target protein (also known as twilight-zone protein, sequence identity with available templates is less than 30 %), the protein structure prediction has to be initiated from scratch. Traditionally, twilight-zone proteins can be predicted via threading or *ab initio* method. Based on the current trend, combination of different methods brings an improved success in the prediction of twilight-zone proteins. In this mini review, the methods, progresses and challenges for the prediction of twilight-zone proteins were discussed.

## Introduction

Specific function and mechanism of a protein can be elucidated from the three dimensional (3D) structure of a protein. The most accurate way to determine a high resolution protein structure is through experimental methods such as X-ray crystallography or nuclear magnetic resonance (NMR) spectroscopy [1, 2]. As of January 2015, the Protein Data Bank (PDB) has over 100,000 deposited protein structures (www.rcsb.org) [3]. With the increasing number of deposited protein structure in PDB, the data is highly beneficial to the computational approach that utilized information from these experimentally-determined structures. Although the number of experimentally-determined protein structures is increasing at an accelerated rate, at the same time, numbers of known protein sequences from genome sequencing projects are increasing. To bridge the protein sequence-structure gap, computational protein 3D structure predictions from its amino acid sequence provide potential solution [4]. Computational protein structure prediction may not be as accurate as experimental method but they often reveal the molecular insight from the predicted structure and could generate hypotheses which are useful to complement the experimental approach and provide fundamental understanding of a protein [5, 6]. Therefore, when the experimentally-determined structures are unavailable, these predictive structures may serve as the starting points to study the protein.

Protein structure prediction is a method of translating the protein sequence into 3D structure through computational algorithms. Computational approaches for prediction protein 3D structures can be generally divided into three categories (comparative modelling, threading and *ab initio* approach). It can also be categorized into template-based (TBM) and template-free (FM) modelling [7, 8]. Comparative modelling and threading method are categorized into TBM as they depend on the availability of a template from solved protein structures [9]. FM (also known as *ab initio* or *de novo* method) is potentially able to predict protein structures without any template [8, 10]. To date, comparative modelling is the most successful and accurate method to produce a reliable structure. However, structure accuracy highly depends on how strong the relationship between target and template (sequence identity >30 %). For closely related protein sequence, sequence similarity usually falls above 30 % [4, 10, 11]. Over 95 % of protein chains with low sequence identity have different structures and this reduced the accuracy of the predictions [12]. As the sequence identity decreases, it leads to the probability of identifying incorrect templates and generating less accurate models with errors in predicted models, such as errors in side-chain packing, distortions and shifts in correctly aligned regions, errors in regions without a template and errors due to template misalignment [13, 14]. In addition, searching for homologous proteins is difficult when the sequence identity is low (also known as the “twilight-zone”), where the sequence identity falls between 10 and 30 % [15]. Thus, when the value is low, sequence identity is generally not a statistical reliable predictor to generate accurate model. Therefore, in such situation threading and *ab initio* method offer an alternative way for protein structure prediction. Previously, twilight zone protein structure prediction focused on the sequence alignment [16–20], the secondary structure prediction [21, 22] as well as the physiochemical properties of amino acids [23–25] to improve the quality of the built model. The scoring function e.g. position specific scoring matrices (PSSMs), Levitt-Gerstein (LG) score [26], LiveBench [27], MaxSub [28], S-score [29], *C*-score [30] where then used to rank the built models. Besides, obtaining an accurate structure for twilight-zone protein is challenging [31]. For this reason, this review will be emphasized on methods for prediction twilight-zone protein from scratch. Focus will be put on threading, *ab initio* and the current trend in protein structure prediction for twilight-zone proteins.

## Threading method

Threading, also known as fold recognition, is used to identify protein templates in PDB bank for similar fold or similar structural motif to the target protein [32]. The concept for threading is similar to comparative modelling but comparative modelling only considers sequence similarity between target protein and template, while protein threading considers the structural information in the template [33]. The critical step of threading is to identify correct template proteins with similar folds to the target protein and make correct alignment [34]. Protein threading compares a target sequence against one or more protein structures to detect and obtain the best compatibility of sequence-structure template pair [1, 33]. They identify best fits of target sequence with the fold template based on the generated alignments and each template is calculated according to different scoring function. Commonly used alignment scores to identify precise target-template alignments include sequence profile-profile alignments (PPA), sequence-structural profile alignments, secondary structure match, hidden-Markov models (HMM) and residue-residue contact [1]. The alignment algorithms are able to search for remotely homologous sequences in the databases. Therefore, even if sequence similarity is low (<30 %), threading method can be used to obtain similar folds or structural motifs for the target sequence. Traditionally, pair-wise comparison is used for matching of single sequences of target and template in the database. PPA, which can be used to detect weak similarities between protein families, is most often used and popular threading approach (successfully used in CASP7 for I-TASSER) [35, 36]. The new threading algorithm MUSTER (Multi-Source ThreadER) showed that accuracy of PPA can be further improved by incorporating various sequence and structure information (e.g. sequence profiles, secondary structure prediction, torsion angles, solvent accessibility and hydrophobic scoring matrix). MUSTER showed a better performance with TM-score 5–6 % higher than PPA in the testing proteins [34].

**Fig. 1:**
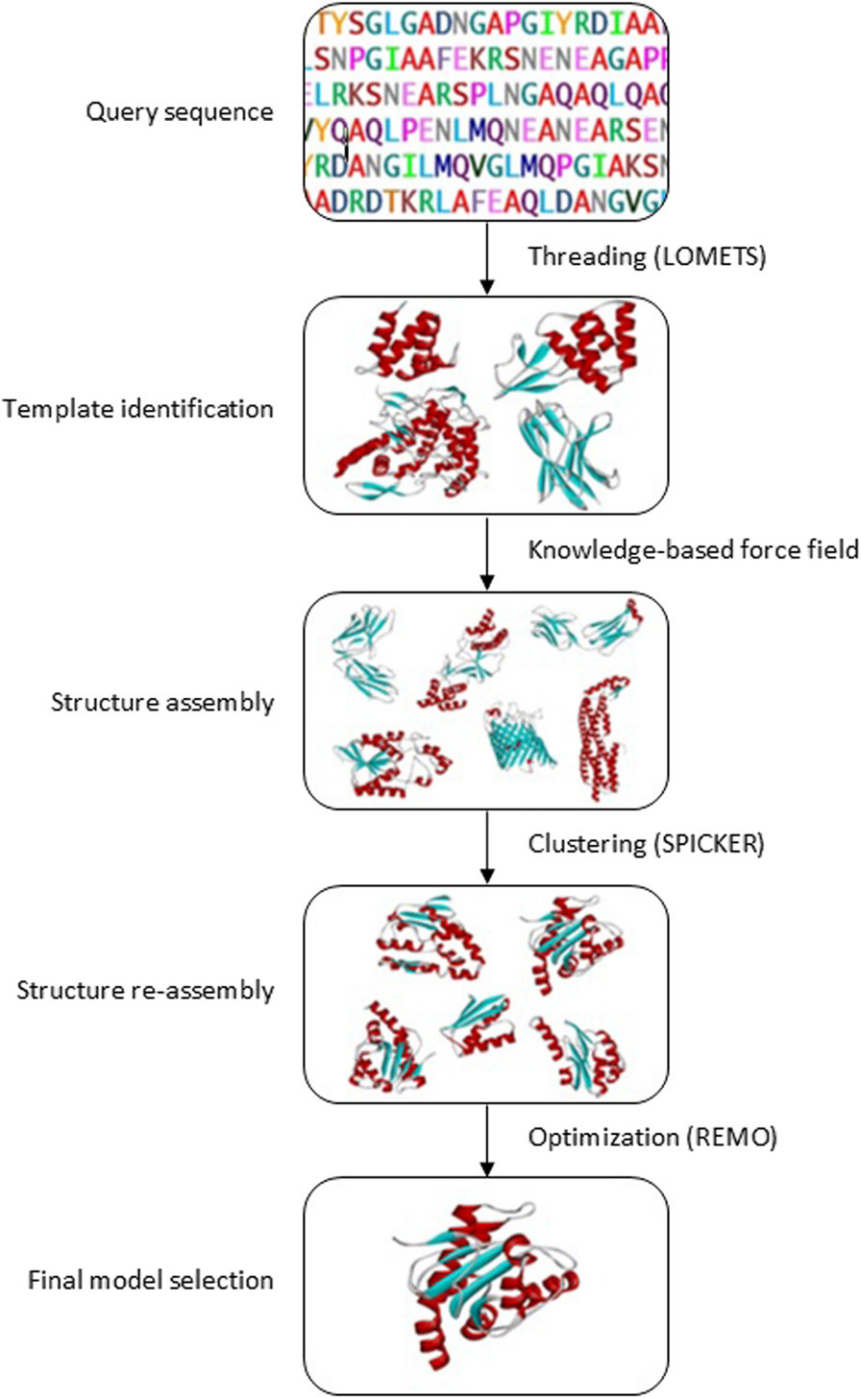
General workflow of I-TASSER for protein structure prediction [30]

The overall procedure for I-TASSER is illustrated in Fig. 1. In general, I-TASSER divided the protein structure prediction into four steps: i) template identification, ii) structural reassembly, iii) model construction and, iv) final model selection. In the first step, the query sequence is threaded through PDB library to identify appropriate fragment using LOMETS algorithm [37]. This will be followed by continuous fragments from the threading alignments are used to assemble full-length models that aligned well, with the unaligned regions (loops/tails) built by *ab initio* modelling [38]. The structure assembly simulations are guided by a knowledge-based force field, including: i) general knowledge-based statistics terms from the PDB, ii) spatial restraints from treading templates, iii) sequence-based contact predictions from SVMSEQ (a support vector machine based residue-residue contact predictor) [37]. After that, fragment assemble simulation is performed again and are clustered by SPICKER [39]. After superposition, all the clustered structures are averaged to obtain the cluster centroids. The final full atomic models are obtained by REMO which builds the full-atomic models from the selected I-TASSER decoys through the optimization of the hydrogen-bonding networks [40]. The forces in REMO protocol include H-bonding, clash/break-amendment, I-TASSER restraints and CHARMM22 potential [37]. For the final top 5 models selection, I-TASSER uses SPICKER to cluster and report up to five models corresponding to the five largest structure clusters. These steps are the essential advantage of TASSER for is its ability to drive the template structures closer to the native than the input templates by ~2–3 Å [41–43]. The confidence level of the predicted model was estimated by *C*-score (Eq. 1).

Eq.1 $$ \mathrm{C}-\mathrm{score}= ln\left(\frac{M}{M_{tot}}\times \frac{1}{RMSD}\times \frac{1}{7}{\displaystyle \sum_{i=1}^7}\frac{Z(i)}{Z_0(i)}\right) $$

TASSER has been tested in CASP6 experiment and emerged as one of the most successful structure prediction methods. It is however, TASSER failed to correctly predict the relative orientation of multiple domain proteins. TASSER’s performance for free modelling targets is yet to be satisfactory as the success rate for non-homologous single-domain proteins is around two thirds [20, 44].

Since no single program has been reported to be outperformed others (within all threading approach), the consensus structure prediction method (meta-server approach) is therefore developed. With this approach, a number of models by multiple threading programs are generated. The idea behind this approach is the models that are generated by different programs are closest to native and less likely to make a common inaccurate prediction [31]. Available meta threading servers include 3D-Jury [45], and LOMETS [46]. 3D-Jury is a meta-server that collects and compares models from various remote protein structure prediction servers [45]. Therefore, the final performance is highly dependent on the inputs from the servers [46]. On the other hand, LOMETS locally installed all threading alignments programs, including PPA, HMM, structural profile and contact-based alignment. This will allow the users to obtain the predictions of all servers quickly compare with 3D-Jury [46]. The meta-server approaches have previously dominated the server prediction in CASP6 experiments. However, in CASP7 experiment, Zhang-Server (I-TASSER) showed better performance than all available meta-server (will be discussed in section ‘Current trend in protein structure prediction’) [47].

## *Ab initio* method

When there is no homologous structure in PDB or the relationship is so distant until it could not be detected by threading, *ab initio* folding is the alternative way to generate protein structure from scratch [1]. This method is termed template-free modelling (FM) (also known as *ab initio* or *de novo* modelling) as it originally referred to methods that based on the first principle laws of physics and chemistry. The idea is also based on Anfinsen’s thermodynamic hypothesis [48]. As above-mentioned, Anfinsen’s hypothesis stated that protein structure prediction depends solely on amino acid sequence [49]. The prerequisite of these modelling methods is that the native structure has the global minimum free energy among all available conformations [32]. Therefore, efficient and reliable algorithm is in need to limit the conformational space in order to minimize the energy function so that the protein is tend to be in its native state [50, 51].

**Fig. 2:**
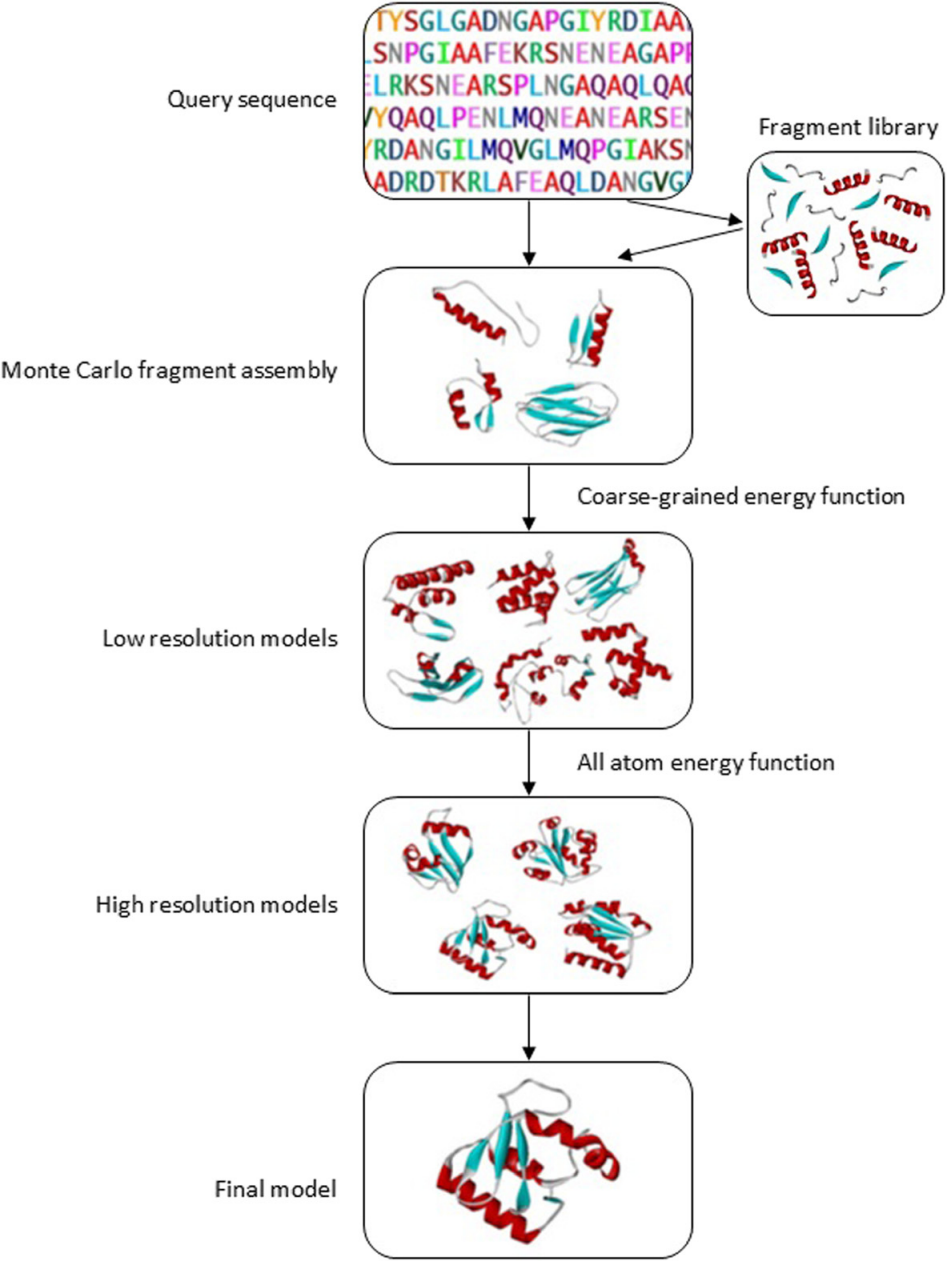
General workflow of Rosetta for protein structure prediction [53]

There have been a variety of methods developed for *ab initio* protein structure generation. The leading approach is the fragment-based assembly method, an idea of Bowie and Eisenberg [11, 51, 52]. Based on this idea, Rosetta [53] was developed and was exceedingly successful in FM as Rosetta is able to produce accurate models nearer to its native structures [54, 55]. Fig. 2 shows the general workflow of Rosetta in protein structure prediction. The idea of fragment-based assembly is that the smaller fragments are restricted to the local structures by most closely related sequence in protein structure database [51, 54]. The lengths of the fragments vary by different programs and the fragment libraries comprise fragments from high-resolution known PDB structures. In Rosetta, fragment libraries of three- and nine- residue were exploited [53]. The original fragment insertion method by Rosetta showed consistent and accurate result compared to other *ab initio* structure predictions in CASP7 [53]. Generation of fragments is important in Rosetta after the completion of secondary structure prediction and it can be done through Robetta server [56, 57]. The program iterates over three- and nine-residue of the sequence and looks for similar sequences from the fragment libraries that Rosetta uses to guide the search of conformational space in predicting protein structures [58]. In Rosetta, method is done by Monte-Carlo algorithm to obtain native condition of protein conformations [53, 59]. Monte-Carlo algorithm generates a structure prediction by randomly inserting fragment predictions into the structure and the energy function is defined as the Bayesian probability of structure/sequence [54]. Bayes statistical theorem is exploited as a scoring function (Eq. 2) [59, 60]:

Eq.2 $$ P\left(\left. structure\right| sequence\right)=P(structure)\times \frac{P\left(\left. sequence\right| structure\right)}{P(sequence)} $$

Rosetta energy functions are classified into two: knowledge-based centroid energy function that uses coarse-grained or low-resolution energy function to treat the side chains as centroids, and the knowledge-based all atom energy function that combines Lennard-Jones potential and a knowledge-based conformation-dependent amino acid internal free energy term [61]. The all atom energy function is more accurate but it is slower comparing with the centroid energy function as the side-chain atoms, van der Waals interaction, hydrogen bonds and pair wise solvation free energy is taking into consideration in all atom energy function. Both coarse-grained and all-atom energy function has been successfully used to predict high resolution protein structures from their sequences.

A newer method, QUARK by Yang Zhang group, successfully predicted models of correct folds for 8 out of 18 proteins with length less than 150 residues in CASP9 [62]. QUARK fragment assembly starts from random conformation that enable it to construct new protein folds from scratch [63]. In QUARK, the models are assembled from small continuous fragments ranged from 1 to 20 residues excised from unrelated proteins by Monte-Carlo simulation [11, 63]. Both Rosetta and QUARK showed the importance of assembling structural models using small fragments by their significant performance in CASP9 [64]. In CASP10, QUARK successfully predicted model with larger size range in FM modelling (>150 residues) [62].

## Current trend in protein structure prediction

In order to improve the performance of *in silico* approaches, the boundaries between the protein structure prediction methods have overlapped due to the integration of the strength of different approaches [31]. Recent CASP experiments demonstrated that composite approaches can achieve additional advantages in structure prediction. Since no single approach can perform better than others for all protein prediction, the emergence of new trend is the combination/hybrid of different protein structure prediction approaches [32, 63].

I-TASSER (Iterative Threading ASSEmbly Refinement) is one notable successful composite approach in the CASP experiments [30]. I-TASSER method is based on the secondary structure enhanced profile-profile threading alignment extended from TASSER algorithm for iterative structure assembly and refinement of protein molecules [43, 65]. I-TASSER retrieves structural template from PDB library through a meta-threading server, termed LOMETS. By year 2010, the online I-TASSER server has generated more than 30,000 full-length structure and function predictions for more than 6000 registered users [30]. I-TASSER can consistently predict correct folds and also high-resolution for small single-domain protein (<120 residues) with a lower computational time (5 CPU hours for I-TASSER and 150 CPU days per target for Rosetta). In CASP7, CASP8, CASP9 and CASP10, I-TASSER was ranked as the best server for protein structure prediction [66].

Butterfoss *et al.* presented blind-structure prediction for three peptoids using the hierarchical combination of Replica Exchange Molecular Dynamics (REMD) simulation and Quantum Mechanical (QM) refinement [67]. They have managed to predict a *N*-acryl peptoid trimer and a cyclic peptoid nonamer with backbone RMSD of only 0.2 and 1.0 Å, respectively. Their findings showed that physical modeling is able to performed *de novo* structure prediction for small peptoid molecules.

In 2013, *Bhageerath*-H Strgen, another homology/*ab initio* hybrid algorithm was developed. The method was tested in CASP9 experiments and showed 93 % of the targets were in the pool of decoys. The results showed that *Bhageerath*-H Strgen is capable of searching the protein fold for near-native conformation. Strategy in *Bhageerath*-H Strgen involved secondary structure prediction, database search for sequence based on the input amino acid sequence, fold recognition, template-target alignment, and template-based modelling by MODELLER [4]. The missing residues with no fragments are modelled using *Bhageerath ab initio* modelling. In their study, they showed that *Bhageerath*-H Strgen performs better than Rosetta and I-TASSER [68].

The Robetta server (http://robetta.bakerlab.org) is an automated server for protein structure and analysis. Protein structures can be generated in the presence or absence of similarity to homologous proteins of known structure. BLAST, PSI-BLAST, FFAS03 or 3D-Jury is used to search for a match to the solved protein structure. When there is a confident match, comparative modelling is used for protein structure prediction. If no match is found, *ab initio* Rosetta fragment insertion method will be used for prediction [58]. In CASP8 experiment, Robetta is ranked as the top 4 best performing groups [69].

## Successes and challenges for twilight-zone protein modelling

The successful rates for twilight-zone protein modelling are increasing over the years with numerous successful examples have been reported. In year 2008, *Leucosporidium antarcticum* antifreeze protein was predicted by comparative modelling, threading and *ab initio* approaches due to low sequence identity. Their study suggests that I-TASSER (*ab initio* approach) is useful for low resolution protein structure prediction for twilight-zone protein.

In 2011, *Chlamydia trachomatis* protein CT296 was determined using both computational method (I-TASSER) and X-ray crystallography method. Despite having no homologs, the result showed that the structure of CT296 predicted by *ab initio* I-TASSER has overall structural similarity (root mean square, RMSD of 2.72 Å for 101/137 residues) to the high-resolution X-ray crystallography structure (1.8 Å). The result showed that I-TASSER is effective to predict accurately twilight-zone protein structures that have no primary sequence homolog with any known proteins [70]. This is an encouraging study for the most challenging twilight-zone protein modelling in protein structure prediction.

Successes in the structure prediction for gas vesicle protein GvpA from haloarchaeon *Haloferax mediterranei* have also been reported. The protein structure was predicted by Strunk *et al.*, and Ezzeldin *et al.*, in year 2011 and 2012 respectively [71, 72]. The structure prediction was first carried out by Strunk and colleague via *ab initio* approach (Rosetta). The predicted structure suggested that GvpA possessed two α-helices and two β-strands. The secondary structure elements (α- β- β- α) is similar with the NMR structures obtained for GvpA protein from cyanobacterium *Anabaena flos-aquae* [73]. Mutation in α-helix and β-turn affected the ability to form gas vesicle. This *in vivo* data on GvpA mutants support the major structural features from the proposed structures.

In the subsequent year, Ezzeldin and colleagues predicted GvpA protein from *Halobacterium sp. NRC-1* with computational comparative modelling (by MODELLER and SCRATCH), threading (by I-TASSER) and *ab initio* modelling (by Rosetta) [72]. All the predicted structures were equilibrated through molecular dynamics (MD) simulation. Average MM-PBSA energy and standard deviation were calculated and ranked. From the comparison of the top ranked predicted structures and an earlier model proposed by Strunk *et al.*, it showed that two sequences possess 93 % identity despite of belonging to different organisms [71]. Furthermore, the structures possessed an α- β- β- α secondary structure, in agreement with previous experimental data and their secondary structure prediction [72]. The predicted model thus support the hypothesis that homologous sequences synthesized by different organisms should exhibit similar structures [72].

Another research in year 2014 was the structure prediction of *Bm*R1 protein from *Brugia malayi*. In the study, the *Bm*R1 protein (206 residues) was modelled via comparative modelling, threading and *ab initio* approaches. The predicted models were evaluated and compared. Based on the model evaluation, the *ab initio* approaches by Rosetta outperformed others method with a quality and reliable structure from structure validation and evaluations [74].

Despite the rapid progress in structure prediction, there are still significant challenges in the current method [32]. As demonstrated in the CASP experiments, the successful of twilight-zone protein modelling via FM is only limited to small protein below 100 residues [63]. With increasing protein size, the conformational space will also increase proportionally. As mention earlier, it is important to limit conformational space in order to obtain lowest free energy. In CASP 10, QUARK successfully predicted two FM targets with length >150 residues [62]. Although there are successful predictions for twilight-zone protein, there is still a need for a consistent successful rate. For example, in spite of the reported successful cases, the QUARK program has difficulty to consistently assemble the correct protein structures with length >100–120 residues from scratch [63, 75].

Another challenge in twilight-zone protein is to distinguish the correct distantly related proteins from unrelated proteins. The accuracy of comparative modelling is highly dependent on the sequence similarity between the target sequence and template. For closely related protein, sequence similarity usually above 30 % [4, 10]. When the sequence similarity decreases, probability of getting a reliable structure decreases. For this reason, the algorithms and programs to identify correct templates from related proteins play a significant role. Although various template searching algorithms are available online, efficient and consistent template detection is still essential especially for distantly related protein sequences.

## Conclusion and future direction

The elucidation of a protein structure is vital in order to aid the understanding of the biological roles of it in living cells. Comparative modelling can generate high resolution model when evolutionary related homologous templates are identified. The structure of a query protein from different evolutionary origin can be predicted by threading method to recognize folds similar to query. A query must be built from scratch by *ab initio* modelling when no structurally related proteins were found in the template database. Here, we have presented a general review on twilight-zone protein structure prediction from the point of view in both threading and *ab initio* approaches. Although each method reported successful predictions, the composite approaches from threading, *ab initio* and other various methods have showed marked improvement compared to the single method alone. The bottleneck of the twilight zone protein modelling is that the success/accuracy rate is decreased when the protein size is increased. Significant challenges remain in distant-homology identification and refinement. Compounded by the complexity of structure prediction is that about one tenth of proteins are disordered for their physiochemical roles. Therefore, the development of a reliable, efficient and consistent algorithm in fold-recognition and refinement would influence for accuracy in the prediction of twilight-zone proteins.
